# Modification of Weak Localization in Metallic Thin Films Due to the Adsorption of Chiral Molecules

**DOI:** 10.1021/acs.jpclett.3c00702

**Published:** 2023-05-22

**Authors:** Meital Ozeri, Jiahui Xu, Gilad Bauer, Linde A. B. Olde Olthof, Graham Kimbell, Angela Wittmann, Shira Yochelis, Jonas Fransson, Jason W. A. Robinson, Yossi Paltiel, Oded Millo

**Affiliations:** † Racah Institute of Physics and the Center for Nanoscience and Nanotechnology, The Hebrew University of Jerusalem, Jerusalem 9190401, Israel; ‡ Department of Materials Science & Metallurgy, University of Cambridge, 27 Charles Babbage Road, Cambridge CB3 0FS, United Kingdom; § Applied Physics Department and the Center for Nanoscience and Nanotechnology, The Hebrew University of Jerusalem, Jerusalem 9190401, Israel; ∥ Institute of Physics, Johannes Gutenberg University Mainz, Staudingerweg 7, Mainz 55128, Germany; ⊥ Department of Physics and Astronomy, Uppsala University, Box 516, Uppsala 75120, Sweden

## Abstract

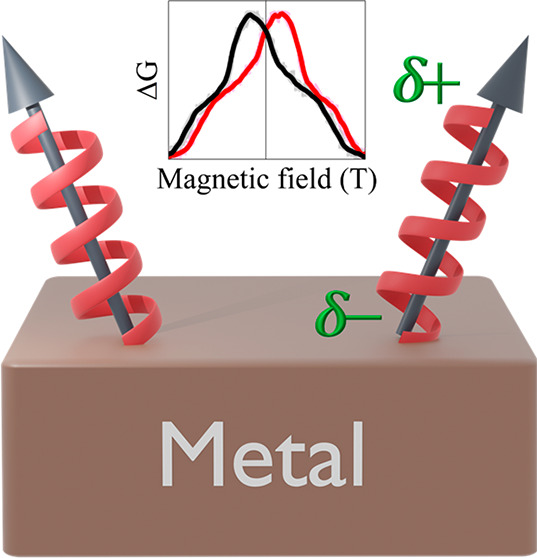

We perform low-temperature magneto-conductance measurements on Cu and Au thin films with adsorbed chiral molecules and investigate their phase-coherent transport properties. Upon adsorption of chiral molecules, the spin–orbit coupling strength in Cu decreases and the Au films become ferromagnetic as evident from weak localization and antilocalization data. A theoretical model indicates that anisotropy in the molecular tilt angles, provided that the chiral molecules act as magnetic moments, induces a nonvanishing magnetic exchange interaction, causing changes in the spin–orbit coupling strength in Cu and Au. Our work adds a new viewpoint to the plethora of unique phenomena emerging from chiral molecule adsorption on materials.

The spin-filtering ability of chiral molecules was discovered over two decades ago and termed the chiral-induced spin-selectivity (CISS) effect.^1^ When an electron passes through a chiral molecule, one spin state is preferably transferred through one enantiomer while the other spin state preferably passes through the opposite enantiomer. The effect was first observed in photoelectron transmission through chiral molecules^2^ and later via electrical transport measurements.^3,4^ Spin-filtering efficiencies that exceed 90% have been demonstrated.^5^ Motivated by these effects, a number of theoretical models^6−19^ were put forward to rationalize the CISS effect, demonstrating that the helical structure of the chiral molecules and their spin–orbit coupling (SOC) are key to their underlying spin-filtering properties.

The adsorption of chiral molecules onto the surface of a material can dramatically modify its electromagnetic properties. For example, thin ferromagnetic films can be magnetized by the adsorption of chiral molecules. The magnetization orientation depends on the handedness of the chiral molecules,^20^ and the magnetization angle corresponds to the tilt angle of the molecules.^21^ Another example is that when chiral molecules are sparsely adsorbed on a conventional (s-wave spin-singlet) superconductor, in-gap states similar to magnetic-impurity-related Yu–Shiba–Rusinov states appear in the density of states, with a zero-bias conductance peak emerging with increasing adsorption density.^22^ Related to these results, Volosniev et al.^23^ showed theoretically that, in a one-dimensional system, an interplay between SOC and a basic dissipative process (frictional dissipation) might explain the pronounced effect of chirality on the spin distribution and transport through chiral molecules. In particular, their model accounts for the appearance of a static magnetic dipole in a chiral molecule upon adsorption. The magnetic dipole of the adsorbed molecules can explain the induced Yu–Shiba–Rusinov states on the surface of a chiral-molecule-adsorbed superconductor.^22^ Applying a different approach, Fransson et al.^24^ demonstrated that vibrationally assisted charge redistribution in chiral molecules coupled to a nonmagnetic metal can give rise to spin polarization due to chiral-induced charge–spin separation.

One fundamental issue that has not been addressed so far is the effect of chiral molecule adsorption on phase-coherent transport properties within a normal (i.e., nonmagnetic, nonsuperconducting) metal. This can provide a complementary method to examine how adsorbed chiral molecules modify the SOC and magnetic properties of the substrate onto which they are adsorbed. Motivated by these questions, here we investigate the manner in which the adsorption of chiral molecules modifies the low-temperature magneto-conductance of Cu and Au thin films. We find that chiral adsorption increases the SOC scattering length in Cu and that Au thin films become weakly ferromagnetic.

SOC enables spin currents to flow in an electronic structure, expressed via the spin-current operator *j*_*s*_ ≈ tr[ψ^+^σ∇ψ – (∇ψ^+^)σψ], where ψ (ψ^+^) denotes the annihilation (creation) spinor and σ is the vector of Pauli matrices. The local variations of the expectation value of this operator are nonvanishing for configurations in which spin symmetry is locally broken, e.g., by SOC. However, spin currents can also be induced between local magnetic moments in a magnetically noncollinear arrangement.^25^ Such spin currents can be described through a Dzyaloshinskii–Moriya-like interaction^26^ and hence can be interpreted as an induced contribution to the SOC. It is therefore relevant to investigate materials, systems, and devices which exhibit such an effective SOC.

The SOC strength in a metal can be characterized by the spin–orbit length extracted from low-temperature magneto-conductance curves. In a metallic and diffusive system at low temperatures, conduction electrons can be scattered without losing phase coherence over large distances relative to the electron mean free path length. In this quantum diffusive regime, the interference between coherently backscattered partial waves, which is otherwise negligible, can no longer be ignored. The correction to the classical conductance at zero magnetic field can be negative (due to constructive interference between time-reversed backscattered paths) or positive (due to deconstructive interference), depending on the SOC strength in the metal.^27,28^ In the presence of a strong SOC, the correction to the conductance (at zero magnetic field) is positive and the effect is termed weak antilocalization.^27,29^ For weak SOC relative to the phase-coherence length, the correction (at zero magnetic field) is negative so that conduction electrons are more likely to backscatter and become more localized, so the effect is termed weak localization. Both weak localization and weak antilocalization are suppressed when a magnetic field is applied normal to the plane of the closed paths (i.e., normal to the metal surface), since the two time-reversed paths each gain an opposite additional Berry phase that destroys the interference. Consequently, weak localization and antilocalization manifest themselves through positive and negative magneto-conductance slopes, respectively. In the case of intermediate SOC, the magneto-conductance first decreases to a minimal value and then starts increasing, where the minimum signifies the crossover from the SOC-dominated to the inelastic-scattering-dominated regimes. The magneto-conductance can be described by the Hikami–Larkin–Nagaoka (HLN) formalism,^27,30^ dictating that the change in conductance, Δ*G*, is given by
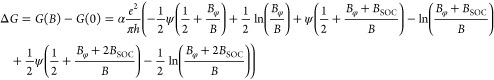
1where *G* is the conductance, *e* is the electron charge, *h* is Planck’s constant, and ψ is the digamma function. *B* is the applied magnetic field,  and  (ℏ is *h* divided by 2π) are the characteristic magnetic decoherence fields related to the phase-coherence length, *l*_φ_, associated mainly with electron–phonon and electron–electron scatterings and the spin–orbit length, *l*_SOC_, which characterizes the SOC strength. At low temperatures, when spin–orbit scattering dominates, *l*_SOC_ matches the spin-diffusion length,^31^ which is the average distance an electron diffuses between spin-flip events. The prefactor α (unitless) depends on the effective number of conduction channels in the material.

We investigate Cu and Au thin films (∼10 nm thick) with a layer of chiral or achiral molecules adsorbed onto their surfaces (see Methods section). The “normalized” magneto-conductance (divided by *e*^2^*/πh*, see eq 1) of a pristine Cu film at different temperatures is plotted in Figure 1a. The weak localization and antilocalization effects are clearly visible in the magneto-conductance curves, and the effects diminish with increasing temperature, as expected. The HLN formalism fits the data very well (green curves in Figure 1).

**Figure 1:**
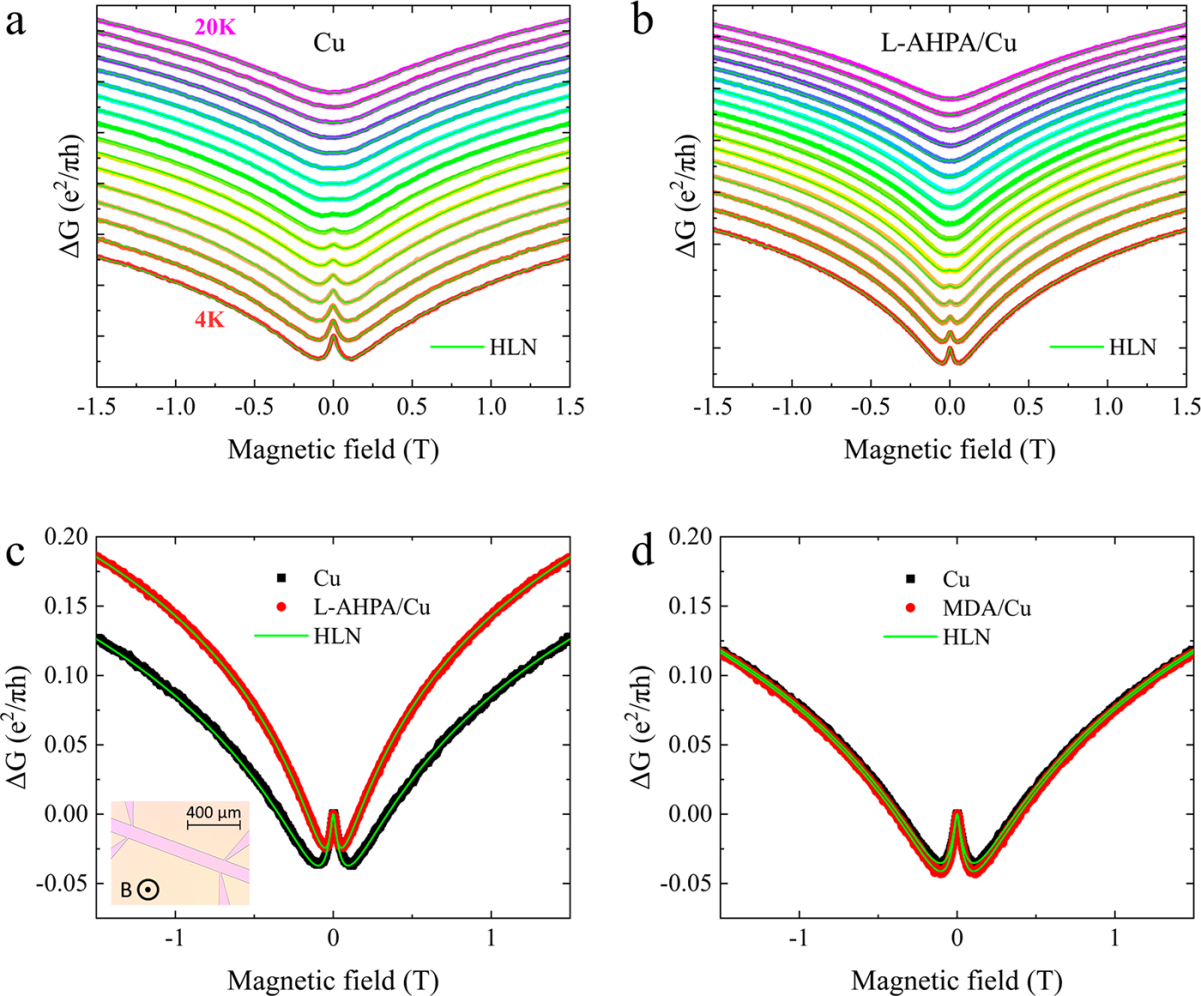
Normalized magneto-conductance of a 10-nm-thick Cu film (a) before (pristine film) and (b) after the adsorption of chiral l-AHPA molecules, at various temperatures (from the bottom plot to the top, the temperatures range from 4 to 20 K in 1 K increments). Each curve is manually shifted for clarity. (c, d) Magneto-conductance at 4 K of a 10-nm-thick Cu film with (red circles) and without (black squares) adsorbed (c) chiral l-AHPA molecules and (d) achiral MDA molecules. Light-green curves are fits to the HLN formalism (eq 1). Inset: optical microscope photograph of the Cu sample showing the hall-bar pattern.

After the adsorption of chiral molecules, the two minima in the magneto-conductance (crossover from the SOC-dominated to the inelastic-scattering-dominated regimes) shift to lower magnetic fields (Figure 1b). This indicates a decrease in the SOC strength in the metal after the adsorption, which is corroborated by the spin–orbit lengths extracted from the HLN fits, as presented in Figure 2. The magneto-conductance curves at 4 K before and after adsorption of the chiral (l-AHPA) or achiral (MDA) molecules are plotted in Figure 1c and 1d, respectively. After the chiral adsorption, we observe a significant decrease in the crossover field, indicating reduced SOC, an effect which is not seen after the achiral (MDA) adsorption.

**Figure 2:**
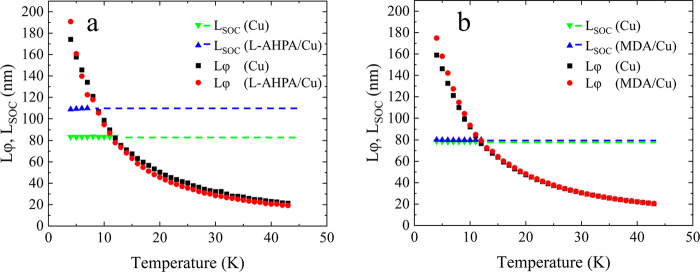
Temperature dependence of the spin–orbit length, *l*_SOC_, and the phase-coherence length, *l*_φ_, in Cu without (pristine film) and with adsorbed (a) chiral or (b) achiral molecules, as extracted from fitting the magneto-conductance curve to the HLN formalism. The dashed lines present the average spin–orbit length before (green) and after (blue) the adsorption of molecules, used in fitting the magneto-conductance above the temperature at which *l*_SOC_ ≈ *l*_φ_, as explained in the Methods section.

The temperature dependence of *l*_SOC_ and *l*_φ_, determined from magneto-conductance curves through the HLN formalism, is presented in Figure 2. Evidently, the adsorption of either chiral or achiral molecules does not significantly change the phase coherence length, *l*_φ_ (black and red symbols in Figure 2 for the pristine and adsorbed films, respectively). This is possibly due to the relatively high energies involved in exciting vibrational or electronic levels in the molecules, thus hindering inelastic scattering from the molecules that increase the phase-breaking rate. On the other hand, the adsorption of chiral molecules increases the spin–orbit length by about 30%, from ∼83 to ∼109 nm (green and blue symbols in Figure 2 for the pristine and adsorbed films, respectively). This signifies an increase in the spin-diffusion length and thus weaker SOC. In contrast, the adsorption of the achiral molecules hardly affects the spin–orbit length (∼78 and ∼80 nm before and after adsorption, respectively), despite the same bonding chemistry and comparable physical properties of the two types of molecules (see Methods section).

For the Au films, the magneto-conductance exhibits only weak antilocalization (Figure 3a) due to the relatively high SOC of the metal. Magneto-conductance curves at 1.7 K on a 7-nm-thick Au film without and with adsorbed l-AHPA chiral molecules are plotted in Figure 3b and 3c, respectively. A clear hysteresis of about 10 mT is observed in the magneto-conductance of the chiral-molecule-adsorbed Au. The hysteresis is distinct from the trivial, weaker, hysteresis seen in Figure 3b for pristine Au, which is an artifact of residual fields in the superconducting solenoid. The induced ferromagnetism can also explain the different shape of the postadsorption magneto-conductance curves. Our results are consistent with a previous observation of chiral-induced magnetization in a gold film via superconducting quantum interference device magnetometry.^32^ We note that the chiral molecules are adsorbed on the Au film via a thiol–Au bond, which was shown to induce paramagnetism^33^ or ferromagnetism^34,35^ in Au even for achiral molecules. However, as evident from the data in Figure 3, the hysteresis in the chiral-adsorbed Au is stronger than that in the achiral-adsorbed Au.

**Figure 3:**
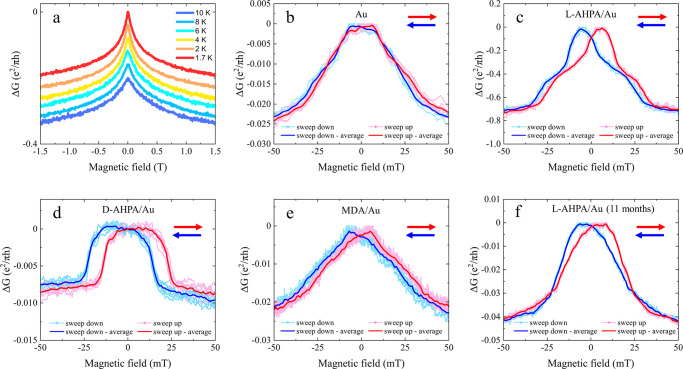
Magneto-conductance curves of Au (7 nm thick) with and without adsorbed molecules: (a) Temperatures dependence of magneto-conductance in pristine Au exhibiting weak antilocalization. Magneto-conductance of (b) pristine Au at 1.7 K, Au adsorbed with (c) l-AHPA chiral molecules at 1.7 K, (d) d-AHPA chiral molecules at 2 K, (e) MDA achiral molecules at 2 K, and (f) l-AHPA adsorbed 11 months prior to the measurement, at 2 K. Pink (and light-blue) curves were measured while the magnetic field changed from negative to positive (positive to negative) values. Seven repetitions are presented for each sweep direction. Red and blue curves represent the moving window average (width 1 mT) of the pink and light-blue curves, respectively.

Hysteresis is also seen after adsorbing d-AHPA molecules, with the enantiomer having an opposite chirality to that of the l-AHPA molecules, as presented in Figure 3d. As discussed in detail below, these effects stem from the fact that the chiral molecules act as magnetic moments when adsorbed on the metals. Since chirality reversal only switches the polarity of the magnetic moments, both enantiomers should induce a similar hysteresis effect in the magneto-conductance curves, as seen in Figure 3. For the same reason, chirality reversal should also reverse the net magnetic field originating from the molecules. However, this field is much too small to be detected in our measurement due to the relatively low density of adsorbed molecules (<10^13^ molecules per cm^2^). This is in contrast to our previous studies of chiral molecules adsorbed onto ferromagnetic films,^20,21,36^ where the spin-exchange forces originating from the molecules flip magnetic domains in the ferromagnet, leading to a detectable magnetic field dependence on chirality reversal.

The adsorption of thiolated achiral MDA molecules also induces magnetism in the gold film (Figure 3e), although it is weaker than that of the chiral-adsorbed Au. This indicates that the thiol–Au bond of the molecules to the surface of the gold makes some contribution to the resulting magnetism, but the chirality of the molecule itself significantly enhances the effect. The magnetization of gold due to the adsorption of chiral molecules is a remarkably stable effect, as demonstrated by Figure 3f that depicts hysteresis in a different Au film with l-AHPA molecules that were adsorbed 11 months prior to the measurement.

As noted in the introduction, adsorbed chiral molecules can have static magnetic properties,^20,22,23,37^ owing to the CISS effect generating spin polarization across the molecules. Additionally, the adsorbed molecular layer has regions with different molecular azimuthal angles (as illustrated in Figure 4a), as detailed in the Methods section. Therefore, we model our system as noncollinear magnetic moments on the surface of a metal that induce a nonvanishing Dzyaloshinskii–Moriya interaction (DMI). While DMI is typically based on spin–orbit interactions and broken inversion symmetry, it was also shown^25,26^ that it can arise in electronic structures with charge and spin currents. The currents may be regarded as conceptually analogous to the orbital motion of an electron, but constituted of itinerant electrons circulating the configuration of noncollinear magnetic moments. The driving force for this asymmetric anisotropic indirect exchange is provided by the noncollinear magnetic moments themselves, mediated by the electron density surrounding the moments. Here we apply this theory under the aforementioned premise that magnetic moments are generated by the chiral molecules when interfaced with the metal.

**Figure 4:**
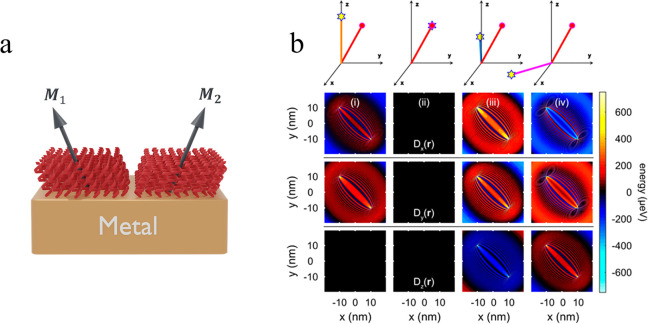
(a) Illustration of a domain boundary between two regions with different azimuthal angles. The molecular tilt directions in the domains are denoted by ***M***_1_ and ***M***_2_, as in eq 2. (b) Simulated induced DMI between two magnetic moments for different configurations, schematically displayed in the top row, where the different components of the interaction vector ***D*** (*D*_*x*_, *D*_*y*_, and *D*_*z*_) are plotted from the second to fourth (bottom) rows, respectively. The moments are located at (−10, 10) nm and (10, −10) nm on a two-dimensional electron gas with a parabolic band and a band bottom −0.5 eV below the Fermi energy.

It is well known that magnetic moments that are embedded in an electronic structure, such as a metallic film, interact indirectly through the electronic medium. (See Fransson et al.^25^ for a general treatment.) This interaction comprises isotropic and anisotropic contributions, for which an antisymmetric anisotropy has the functional form of the DMI, ***D***·(***M***_1_ × ***M***_2_), where ***M***_*i*_, *i* = 1, 2 represents local magnetic moments located at ***r***_*i*_, interacting via ***D***. These interactions are present in any electronic structure which has intrinsic SOC.

If the local magnetic moments are noncollinear, then an additional contribution to this interaction emerges, stemming from the presence of the moments themselves.^26^ The induced DMI (***D***) between the two magnetic moments ***M***_*i*_ coupled to metallic surface states can be written as
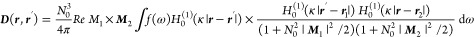
2where *H*_0_^(1)^(*x*) is the Hankel function of the first kind, *f*(ω) denotes the Fermi–Dirac distribution function, , where *E*_F_ is the Fermi energy, and *N*_0_ = *m*_e_/ℏ^2^, with *m*_e_ being the electronic mass.

This expression demonstrates that noncollinearity of the magnetic moments ensures a nonvanishing DMI, as depicted in Figure 4, even in the absence of intrinsic spin–orbit interactions. Therefore, the induced DMI may either enhance or weaken the DMI that arises from the intrinsic spin–orbit interactions.

Assuming a configuration of two effective magnetic moments, as illustrated in Figure 4a, the induced DMI is plotted in Figure 4b for four different configurations, visualized in the top panel. The variations of *D*_*x*_(*r*), *Dy*(*r*), and *D*_*z*_(*r*) as the magnetic configuration is changed are displayed in the three rows in Figure 4b, from top to bottom, respectively. In the simulations presented in Figure 4b, the distance between the moments is about 28 nm. The energy scale of the induced interaction depends reciprocally on |***r***_1_ – ***r***_2_|^2^, which means that the interaction strength is expected to increase by an order of magnitude for each 3 nm decrease in the distance between the moments. The calculated interaction energy is comparable to the typical estimates of the Rashba SOC in both Cu and Au.^38^

In our system, noncollinear spin configurations form on Cu as different domains of adsorbed chiral molecules that have different azimuthal tilt angles (Figure 4a). This may explain the reduced SOC in copper after the adsorption of chiral molecules as seen in Figure 2a.

For the case of Au, the arrangement of the chiral molecules may result in changes in the effective SOC, due to the DMI, that can sustain a weak ferromagnetic state.^26^ In this sense, it is possible that the adsorbed chiral molecules on Au facilitate the emergence of the observed signatures of ferromagnetism. It is known that Au has a much stronger SOC than Cu, which may be the reason that the signatures of ferromagnetism are observed only in Au and not in Cu.

To conclude, we find further experimental data suggesting that chiral molecules act as magnetic dipoles upon adsorption due to spin polarization across the molecules. The adsorption of chiral molecules on the surface of Cu thin films modifies the SOC strength in the metal. Assuming a nonideal adsorption, providing domain boundaries between regions of varying molecular tilt angles, a theoretical model can explain the change in spin–orbit coupling due to the adsorption of such molecules. The model assumes that the chiral molecules behave as magnetic moments coupled to the Cu, an assumption based on previous observations that such molecules were shown to have magnetic-like effects when adsorbed onto materials.^20,22,23^ Thus, the model does not apply to achiral molecules which indeed do not affect the spin–orbit coupling in Cu.

When adsorbed onto gold thin films, chiral molecules induce ferromagnetism in diamagnetic Au, as demonstrated by a hysteretic weak antilocalization signal. Some part of the magnetization can be associated with the thiol–Au bond between the molecules and the Au; however, the effect is more pronounced when the molecules are not only thiolated but also chiral. This is in agreement with a previous observation^32^ of ferromagnetism in Au thin films induced by chiral molecule adsorption. The effect is extremely stable, with the Au retaining its ferromagnetic properties for over 11 months since the chiral adsorption. The different effects of chiral adsorption on Cu and Au may be due to the different SOC strengths of the metals, being much higher in Au than in Cu.

Both results point to changes in the metals after the adsorption of chiral molecules and the importance of the SOC coupling of the substrate when modeling a chiral molecule adsorbed system.

## Methods

Thin films of Cu or Au were deposited via thermal evaporation and patterned by optical lithography into a Hall bar with a 0.1-mm-wide conducting channel and a 0.8 mm separation between the potential probes. (See a sample photograph as the inset in Figure 1c.) The molecules are adsorbed onto the Au or onto the native Cu oxide layer on the Cu via a self-assembly process. During the self-assembly process, molecules spontaneously adsorb to the surface of the films in an ordered fashion. The chiral molecules used are α-helix-polyalanine (l- or d-AHPA for the right- and left-handed enantiomers), and the achiral molecules are 12-mercaptododecanoic acid (MDA). The molecules (both chiral and achiral) have a carboxyl end group and a thiol end group. Both end groups can bond to the Cu oxide to form a self-assembled layer, although the thiol end group has a higher affinity for the Cu oxide.^39^ On Au, the molecules bond to the surface via their thiol end groups. The strong thiol–Au bond is known to facilitate stable self-assembled monolayers.^40^

The MDA achiral molecules were chosen specifically because they have the same end groups, in order to have a comparable electric dipole to that of the chiral molecules used. The MDA molecules were also chosen to have the maximal length that still allows the formation of a self-assembled layer without folding, in order for their length to be comparable to that of the chiral molecules.

The tilt angle of an adsorbed self-assembled molecular layer depends on the chemical nature of the substrate^41^ and on the coverage density.^42^ If the adsorption does not form as an ideal single-crystalline monolayer, then it will likely contain domain boundaries^43−45^ between domains with different azimuthal directions of the same tilt angle.^46^ In this study, the molecules are adsorbed on polycrystalline Au or Cu surfaces. For Au, the molecules are adsorbed overnight in a solution of molecules in ethanol with a concentration of 1 mM, a process that could not be used for the copper films due to rapid surface oxidation. For Cu, the adsorption procedure must be rapid to avoid surface oxidation, so molecules are adsorbed via a drop-cast process which involves dropping a solution of molecules onto the Cu surface. It is unlikely that the assembly process for Cu achieves an ideal monolayer which often requires both a flat surface and long ordering times in solution. Tilt domain boundary “defects” were even observed in alkanethiol self-assembled monolayers on single-crystalline gold.^47^ Additionally, self-assembled l-AHPA molecules (the chiral molecules used in this study) were shown to have varying azimuthal angles when adsorbed on gold.^36,48^ Therefore, the self-assembled layers of molecules in this study certainly contain domains with different azimuthal tilt angles.

On the samples before and after molecular adsorption, temperature-dependent magneto-conductance measurements were performed in a ^4^He pulse-tube cryostat with a superconducting solenoid, using a current source and a voltmeter in a four-point probe configuration.

The values of the spin–orbit and phase-coherence lengths at different temperatures are extracted from the magneto-conductance curves via fits to the HLN formalism (eq 1). We fix the prefactor α according to its value obtained from fits to the magneto-conductance measured at the lowest temperatures. At high-enough temperatures (such that *l*_SOC_*≈ l*_φ_), the spin–orbit length cannot be reliably obtained from the magneto-conductance, which does not change its slope since only paths contributing to weak localization affect it. Consequently, in that higher temperature range, *l*_SOC_ is fixed to the average of its values at the lower temperature range (as it should be temperature-independent) and *l*_φ_ is extracted as the only free parameter. After the pristine metallic films were measured, chiral or achiral molecules were adsorbed onto the surface of the films and the magneto-conductance of the films was measured again.
